# Determination of operation performance indicators of unit for mowing crops with the simultaneous incorporation of their stubble into the soil

**DOI:** 10.1038/s41598-024-66183-x

**Published:** 2024-07-04

**Authors:** Volodymyr Nadykto, Gennadii Golub, Savelii Kukharets, Volodymyr Kyurchev, Oleksandr Skliar, Taras Hutsol, Szymon Glowacki, Tomasz Nurek, Iryna Horetska, Anatoly Yakovenko

**Affiliations:** 1 Department of Operation and Technical Service of Machines, Dmytro Motornyi Tavria State Agrotechnological University, 6 Zhukovskyi Str., 66, Zaporizhzhia, 69-002 Ukraine; 2 Department of Tractors, Automobiles and Bioenergy Resources, National University of Life and Environmental Sciences of Ukraine, Heroev Oborony Str., 15B, Kyiv, 03-040 Ukraine; 3 Department of Mechanical, Energy and Biotechnology Engineering, Agriculture Academy, Vytautas Magnus University, Studentų Str. 11, Akademija, 53362 Kaunas, Lithuania; 4 Department of Mechanics and Agroecosystems Engineering, Polissia National University, Zhytomyr, 10-008 Ukraine; 5 Ukrainian University in Europe-Foundation, Balicka 116, 30-149 Krakow, Poland; 6 Department of Fundamentals of Engineering and Power Engineering, Institute of Mechanical Engineering, Warsaw University of Life Sciences (SGGW), 02-787 Warsaw, Poland; 7 Department of Biosystem Engineering, Institute of Mechanical Engineering, Warsaw University of Life Sciences-SGGW, 02-787 Warsaw, Poland; 8 Department of Agricultural Engineering, Odesa State Agrarian University, Odesa, 65-012 Ukraine

**Keywords:** Two-harvesting method, Windrower, Windrow, Disc harrow, Soil moisture, Laboriousness, Solid Earth sciences, Engineering

## Abstract

When harvesting grain crops and forage grasses using a two-phase method, trailed and/or mounted windrowers are usually used. After their passage, stubble remains on the field, which intensively loses soil moisture under sunlight and wind. To reduce these losses, the stubble, along with the soil, is crushed using disc harrows. Due to the use of two sequentially operating units (harvesting and soil-cultivating), their total operating time increases. This does little to preserve soil moisture in the cultivated field. This article provides an example of a more effective solution to this problem. It consists of using one machine-tractor unit instead of two. The proposed combined unit mows an agricultural crop in one working pass and ensures stubble crushing and incorporation into the top layer of soil. The unit consists of a wheeled tractor with a front hitch linkage, a front windrower and a disc harrow mounted behind the tractor. It has been established that the laboriousness of compiling such a unit, considering the tractor's transformation to reverse, is insignificant and amounts to 1442 person-hours. The use of the new unit assists in reducing soil moisture losses. Over a month, it can reach 4.1–5.2% in absolute terms and 15–45% in relative ones. The combined unit movement velocity should be close to 2.5 m s^−1^ to ensure such a reduction in soil moisture losses. Combining two technological operations performed by one machine-tractor unit does not impair its reliability. At the same time, there is a reduction in processing time for one field by almost half and a decrease in fuel consumption per unit of performed area by 2.25 times.

## Introduction

Very often, grain harvesting is carried out using a two-phase method. Trailed harvesting units include a wheeled tractor and a windrower^1–3^. A particular property of these machine-tractor units (MTUs) is that they are asymmetrical^4^. The consequence of this is a deterioration in the MTU's directional stability, leading to a decline in the quality of its work^5^. This is why trailed harvesting units are used less and less in practice.

Mounted harvesting units are used more often^6–8^. But even after their passage, the stubble of the mown crop in the inter-swath space is exposed to sunlight and wind^9–12^. And this provokes intense soil moisture evaporation^10,13–16^.

The practice of conducting fieldwork involves chopping stubble and incorporating it into the soil no later than 2–3 days after crop harvesting. In reality, this technological operation is performed with a delay. At the same time, its timely implementation allows for soil moisture preservation within 2–6 mm^17^. Based on empirical and mechanistic models, it has been established^18–20^ that each additional 1 cm of soil moisture can increase winter wheat yield by 176 kg ha^−1^.

In our opinion, the crop stubble chopping should be carried out simultaneously with its mowing. In the Ukraine steppe conditions, this can help reduce moisture losses in the soil by 50–100 t ha^−1^ or 5–10 kg m^−2^ per day^21,22^. In addition to preserving moisture, disking the stubble simultaneously with harvesting the crop provokes the germination of weeds in the inter-swath spaces. This makes more efficient their destruction in the future. It should be considered that combining two technological operations (harvesting and tillage) into one can significantly reduce the field's preparation time for its subsequent use.

There are known attempts to combine harvesting and tillage with a unit consisting of a combine harvester and a trailed stubble plough^23^. However, the latter's presence significantly complicates such a harvesting unit movement in reverse when maneuvering on the headland. At the moment, the motion dynamics of this harvesting system have not been studied, which is why neither its diagram nor design parameters have been justified.

Our practical experience suggests that the rear tillage machine should not be trailed but mounted. For this purpose, we have developed a unit consisting of a tractor with a header placed on the front hitch linkage and a disc harrow on the rear^24^. Preliminary studies have established that for better stability and controllability of such unit movement, the header and disc harrow should be attached to the tractor, excluding their ability to turn relative to each other in the horizontal plane. In this case, the tractor can have rear steerable wheels, for which its control post rotates 180° in the cabin.

At the same time, there is no experimental data on the effect of such a harvesting unit design on soil moisture conservation in inter-swath spaces. There is no information on the dependence of this process on the harvesting unit movement velocity. Meanwhile, it has been experimentally established that increasing the disc harrow movement velocity contributes to increased soil crumbling^25–27^. According to research data^28^, it reaches 86–87% when this tillage machine operates at speeds up to 3.61 m s^−1^.

In this study, one unit carried out the technological operations of mowing an agricultural crop into a swath and chopping its stubble along with soil in the inter-swath space. Taking this into account, the purpose of our research, the results of which are presented in this article, is to solve the following problems.

(i) determining the putting together laboriousness of a combined unit consisting of a reversible tractor, a front windrower and a disc harrow;
(ii) establishing the dynamics of changes in soil moisture in the inter-swath space at different velocities of stubble chopping and incorporating it into the soil;
(iii) determining the combined unit's operational and technological performance indicators compared to two other units that mow the crop into swathes and disc the soil separately.

## Materials and methods

### Method for determining the laboriousness of compiling harvesting unit

**Figure 1:**
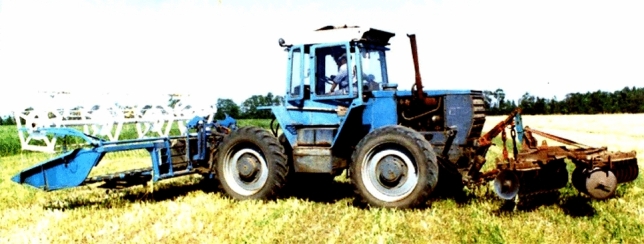
Harvesting unit with reversible tractor movement.

**Figure 2:**
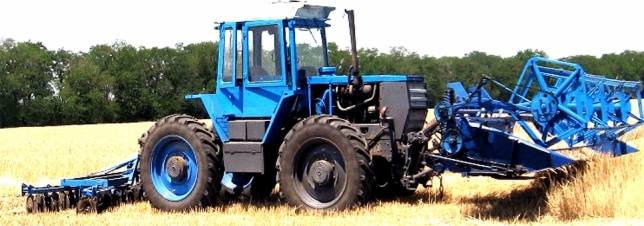
Harvesting unit with direct tractor movement.

The unit consisting of a wheeled tractor XTZ-16131 (Kharkiv, Ukraine), a windrower ZhVN-6 (Berdyansk, Ukraine) and a mounted disc harrow BDN-3 (Ukraine) was taken as a physical object of study. This unit's brief technical description is given in Table 1. The KhTZ-16131 tractor has a reversible control post and a reversible gearbox. As part of the harvesting unit, it can be set to reverse (Fig. 1) and direct (Fig. 2) working stroke.

**Table 1:** Harvesting unit technical characteristics.

Index	Value		
	tractor	Windrower	Harrow
Power engine (kW)	117.6		
Operating weight (kg)	8200	1290	620
Operating width (m)		6.0	3.1
Wheel track (mm)	2100		
Wheelbase (mm)	2860

**Figure 3:**
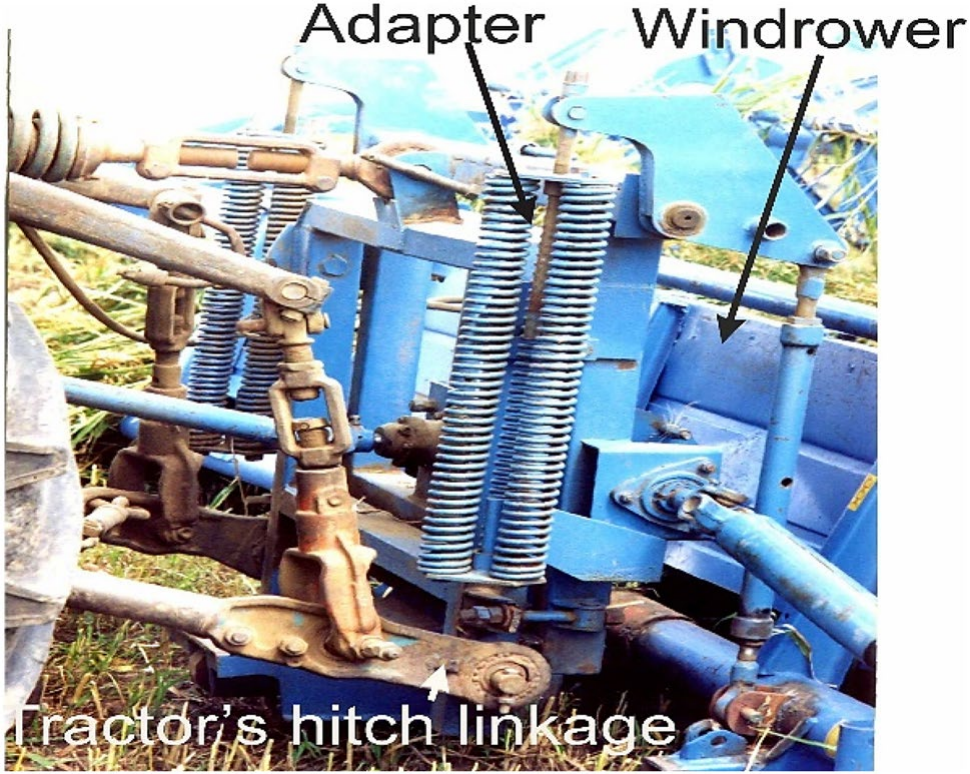
Windrower adapter.

The windrower has a special adapter to attach to the tractor's front three-point hitch linkage (Fig. 3). The need for the tractor to reverse is due to better visibility of the front windrower working devices from the tractor driver's seat. In this case, being closer to the windrower, he can better see its left divider, unloading window and cutting mechanism.

Ultimately, this creates the preconditions for the harvesting unit to perform the technological process without flaws.

The converting laboriousness (${L}_{w}$, person-hour) the tractor from direct to reverse motion, as well as the mounting windrower and a disc harrow on it, was determined from the equation^29^:

1 $$\begin{array}{c}{L}_{w}=\sum_{i=1}^{n}N\cdot {t}_{i},\end{array}$$

where $N$ is the number of persons involved in the preparation of the harvesting unit; $n$ is the number of operations to prepare the unit for operation; $t$ is the time spent on the *i*-th operation, hour. To measure this parameter in three repetitions, we used an electronic stopwatch KHP PC3860 (China), with a measurement error of 0.01 s to register it.

### Method for assessing the impact of the harvesting unit movement velocity on soil moisture

**Figure 4:**
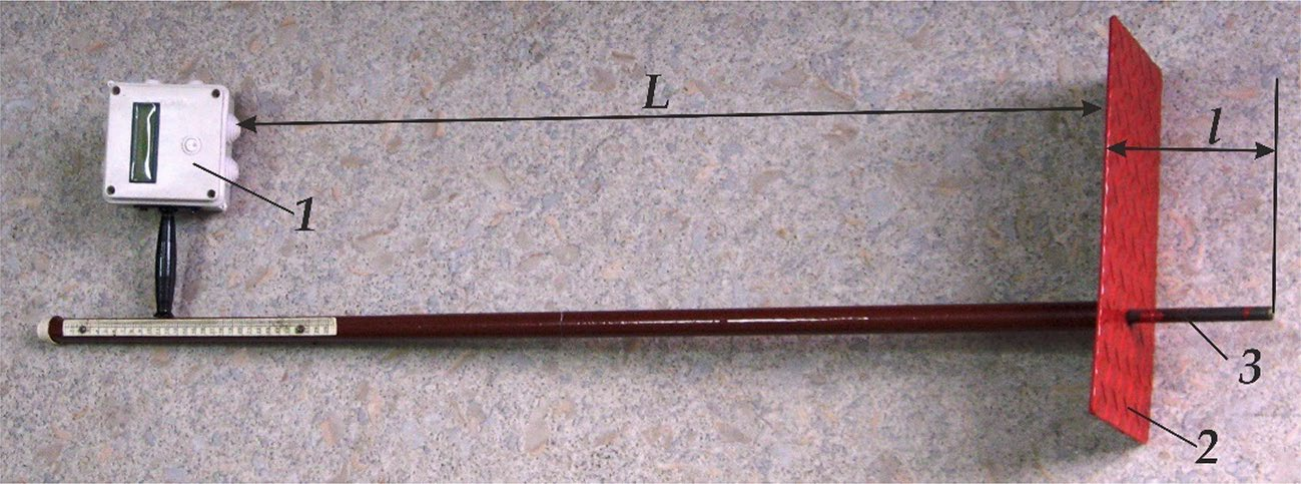
Kit for measuring soil tillage depth: 1—Arduino; 2—support; 3—measuring probe.

The research of the harvesting unit was carried out in two observation periods: (1) July 2021 and (2) August 2021. At the beginning of the first period, the harvesting unit mowed winter wheat into swaths on an area of 3 ha (site 1). The tractor was set to reverse, the header was set to mow the stubble at a height of 15 cm, and the disc harrow was set to disk the stubble to a depth of 6 cm. The same area (3 ha) of winter wheat was swathed in the same field without using a disc harrow (site 2). The XTZ-16131 tractor worked with only one front windrower. After the units' first pass in both sites, the following was determined in duplicate:

I. the time ($t$) for the unit to pass through a test site 200 m long, followed by the calculation of its movement velocity (${V}_{p}$) according to the formula: ${V}_{p}=200\bullet {t}^{-1}$, m s^−1^;
II. soil disking depth in the inter-swath space. The measurement number of this parameter in each repetition was 300. We used a measuring kit based on an ultrasonic sensor HC-SR04 (China) and an Arduino UNO board (Italy) to measure the tillage depth (Fig. 4). During the measurement process, measuring probe 3 was buried into the soil by an amount $l$ until it touched the untreated background. An ultrasonic sensor located in the Arduino block (1) recorded the distance *L*. Since $L=f(l)$, the value of the soil disking depth was reflected on the screen of device 1. The error in measuring the tillage depth with such a kit does not exceed 0.3 cm.

After that, soil moisture in the 7–10 cm layer was measured in each site with a 3–4 days frequency in the inter-swath space. This layer was chosen as a comparative layer because it is untreated after the harvesting unit's passage, cultivating the soil to a depth of 6 cm (site 1) and in the site without disking the inter-swath space (site 2). The instruments and the method used for determining soil moisture are described in detail in the article^30^.

In the second research period (August 2021), they were carried out according to the methodology described above. The difference was that the harvesting unit we studied worked at two different velocity modes. On one of them, the velocity of its movement was set so that it did not exceed 1.94 m s^−1^ (7 km h^−1^). The second mode of the harvesting unit movement was provided to bring the velocity of its movement to a value close to 2.5 m s^−1^ (9.0 km h^−1^). The disc harrow was additionally loaded with a ballast of 200 kg. It was needed to eliminate the influence of harvesting unit movement velocity on the tillage stability in depth in the second movement mode. The ballast value was established by preliminary harvesting unit tests during its movement in the modes (< 1.94 m s^−1^ and ≈ 2.5 m s^−1^).

### Method for operational and technological assessment of the unit's operation

For comparative studies, three machine-tractor units were prepared. The first is the harvesting unit we are considering, in which the XTZ-16131 tractor was set to a straight stroke (Fig. 2). The second unit consisted of the XTZ-16131 tractor and the ZhVN-6 front windrower. The third unit included the XTZ-16131 tractor and the BDT-7 disc harrow (Ukraine). Thus, the first unit mowed an agricultural crop (winter wheat) with simultaneous chopping of stubble and incorporating it into the soil. The second and third units carried out these technological operations separately. First, the second unit cut the wheat into swaths. Then, after picking up and threshing these swaths with a combine harvester, the third unit chopped the wheat stubble with its simultaneous incorporation into the soil.

Each of the units was monitored for three working days. In the observation process, the total time of the working day was recorded. To do this, with an error of ± 5 s, a KHP PC3860 (China) stopwatch was used to record each unit's duration (time) performing operating moves, turns, eliminating technological and technical failures, crossings, etc.

All three units (the investigated and two conventional ones) worked on their sites. The width of each was determined from the equation^29^:

2 $$\begin{array}{c}{C}_{\text{opt}}=\sqrt{16\cdot {R}_{\text{min}}^{2}+2{\cdot B}_{p}\cdot \left[{L}_{o}-2\cdot\text{Integer}\left(\frac{1.1\cdot {R}_{\text{min}}+L+D}{{B}_{p}}\right)\cdot {B}_{p}\right]},\end{array}$$

where ${R}_{\text{min}}$ is the unit's minimum turning radius, m; ${B}_{p}$ is the unit's operating width, m; ${L}_{o}$ is the unit's working stroke length, m; $L$ is the unit's stroke length at the headland, m; $D$ is the unit's kinematical width, m.

The stubble height and the windrower operating width were measured during the unit's operation. The plant height of each harvested crop was determined along the field diagonal with a ruler 1 m long with a measurement error of ± 0.5 cm. The number of such measurements was 300, and the measurement step was 1 m.

**Figure 5:**
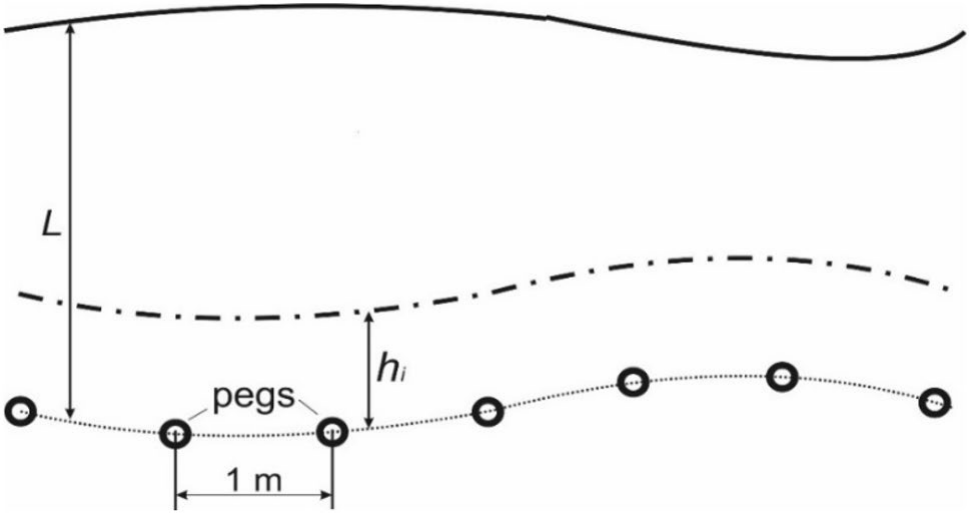
Diagram for determining windrower operating width: (dashed line)—crop array before the harvesting unit passage; (line)—crop array after the harvesting unit passage.

Before the harvesting unit movement, 200 pegs were installed at a distance ${h}_{i}$ from the mowed crop array with a step of 1 m to determine the windrower operating width. After the unit passage, the distance ($L$) from each peg to the wall of the remaining array of mowed crops was measured (Fig. 5).

The operating width of the windrower (${B}_{p}$) was determined from the following equation:

3 $$\begin{array}{c}{B}_{p}=L-{h}_{i}.\end{array}$$

According to the chronometric observations, we calculated the following^29^:

- unit performance for 1 h of main operating time (ha⋅h^−1^):
  4 $$\begin{array}{c}{W}_{m}=0.1\cdot {B}_{p}\cdot {V}_{p};\#\end{array}$$
- operating time utilization rate:
  5 $$\begin{array}{c}\tau =\frac{{T}_{1}}{{T}_{1}+{T}_{2}+{T}_{3}+{T}_{4}};\#\end{array}$$
- 5unit performance for 1 h of total operating time (ha h^−1^):
  6 $$\begin{array}{c}{W}_{o}=0.1\cdot {B}_{p}\cdot {V}_{p}\cdot \tau ;\#\end{array}$$
- stroke factor:
  7 $$\begin{array}{c}\varphi =\frac{{T}_{1}}{{T}_{1}+{T}_{2}};\#\end{array}$$
- technological process safety factor:
  8 $$\begin{array}{c}\gamma =\frac{{T}_{1}}{{T}_{1}+{T}_{3}};\#\end{array}$$
- specific fuel consumption (kg ha^−1^):
  9 $$\begin{array}{c}{G}_{s}=\frac{{G}_{h}}{S}.\#\end{array}$$

In Eqs. (4)–(9), the following notation is adopted: ${T}_{1},{T}_{2},{T}_{3},{T}_{4}$—the time spent by the unit on the performance of working moves, turns, elimination of technological failures and moving to the field, respectively, h; ${G}_{h}$—fuel consumption (kg) per tilled aria $S$ (ha). To measure the ${G}_{h}$ parameter, each tractor was equipped with two DFM 50AK (Bulgaria) sensors with a measurement error that did not exceed 1%.

## Results

### Laboriousness estimation of completing the harvesting unit

It has been established that the laboriousness of preparing the harvesting unit for work is 1.442 person-hours (Table 2). Moreover, at least 90% of it is occupied by converting the tractor to reverse motion. These labour costs can generally be considered insignificant since the tractor is re-equipped and the unit is completed not for one day but for the harvesting period. The latter can last 5–6 days or even more.

**Table 2:** The laboriousness of completing the harvesting unit.

Operation	Laboriousness (person-hour)
Transposition of the tractor for reverse mode	
Transposition of the steering post	0.2500
Transposition of the clutch pedal	0.3500
Transposition of the fuel control lever	0.2000
Transposition of the brakes	0.2500
Transposition of the tractor driver's seat	0.2500
Joining the windrower	
Tractor approach to windrower (from a distance of 5 m)	0.0033
Joining the lower links of the tractor's three-point hitch linkage to the windrower	0.0333
Setting the cardan shaft	0.0237
Joining the high link of the tractor's three-point hitch linkage to the windrower	0.0225
Joining windrower hydraulic system to tractor	0.0072
Blocking the tractor's hitch linkage lower links	0.0450
Joining the disc harrow	
Tractor approach to harrow (from a distance of 5 m)	0.0045
Mounting the harrow to the tractor	0.0025
Total	1.4420

**Figure 6:**
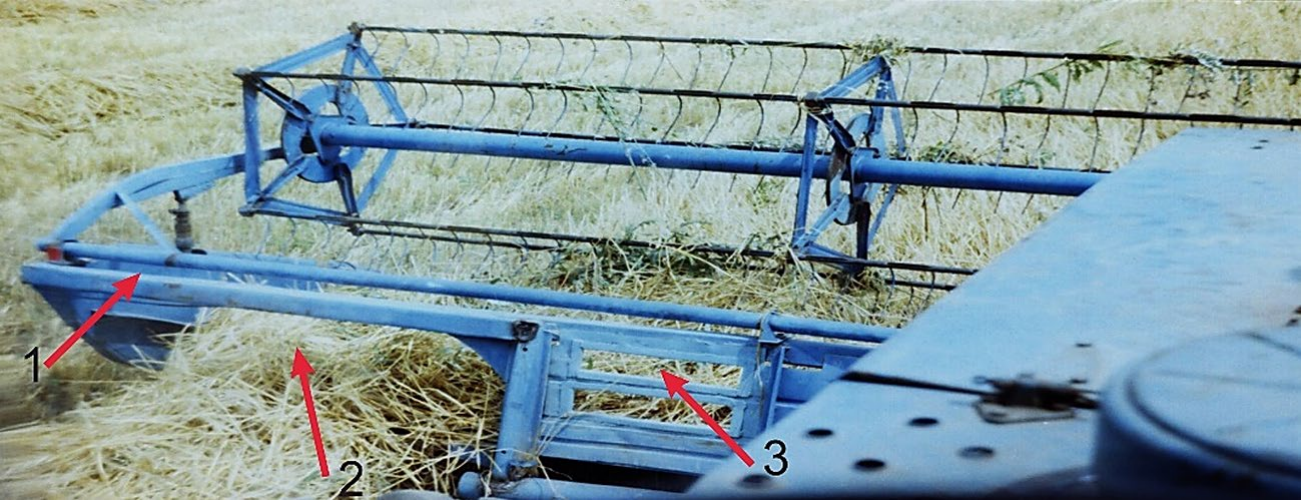
View of the windrower from the tractor driver's seat with the tractor moving forward: 1—left divider; 2—unloading window; 3—viewing window.

Note that not every tractor can be converted to reverse. In this case, its use as part of a harvesting unit provides for the windrower placement on the front three-point hitch linkage. At the same time, the tractor driver is located farther from the front windrower. However, as studies have shown, from his workplace in the cabin, he can see its left divider (pos. 1, Fig. 6) and the unloading window (pos. 2). Moreover, for better visibility, the windrower's cutterbar, a particular viewing window (pos. 3) is made in its frame. All this allows the tractor driver to control the crop mowing process satisfactorily.

### Soil moisture dynamics changes

**Figure 7:**
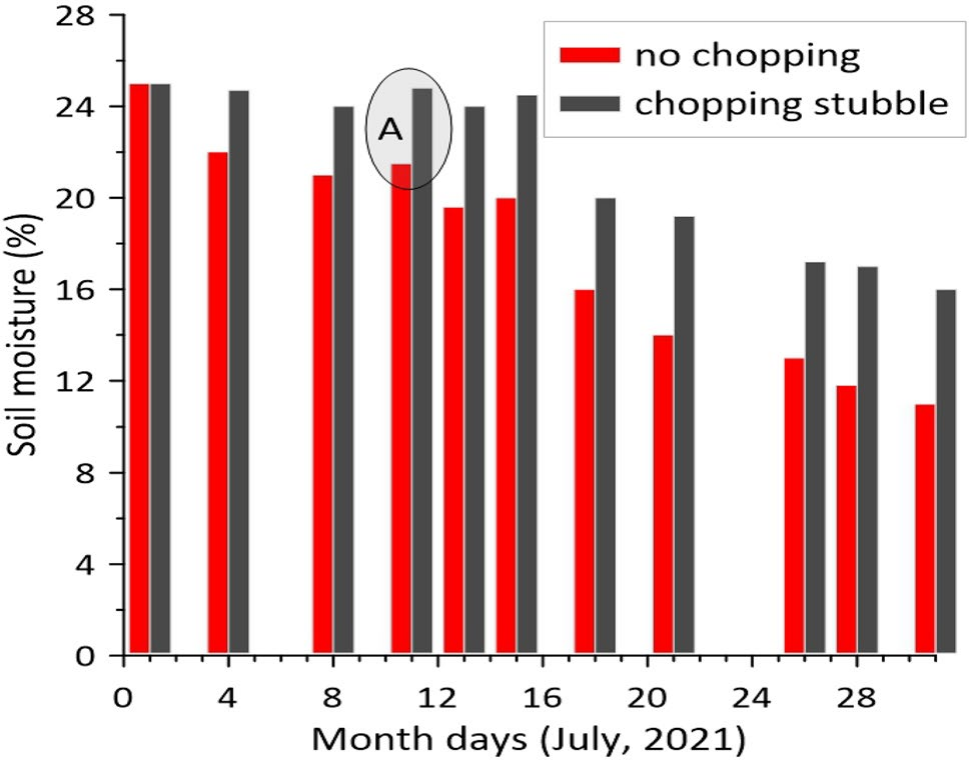
Soil moisture dynamics in the compared inter-windrow sites.

As a result of long-term periodic measurements, the dynamics of the moisture evaporation process from the soil in the crushed and non-crushed spaces between the mowed crop's swathes was obtained (Fig. 7).

**Figure 8:**
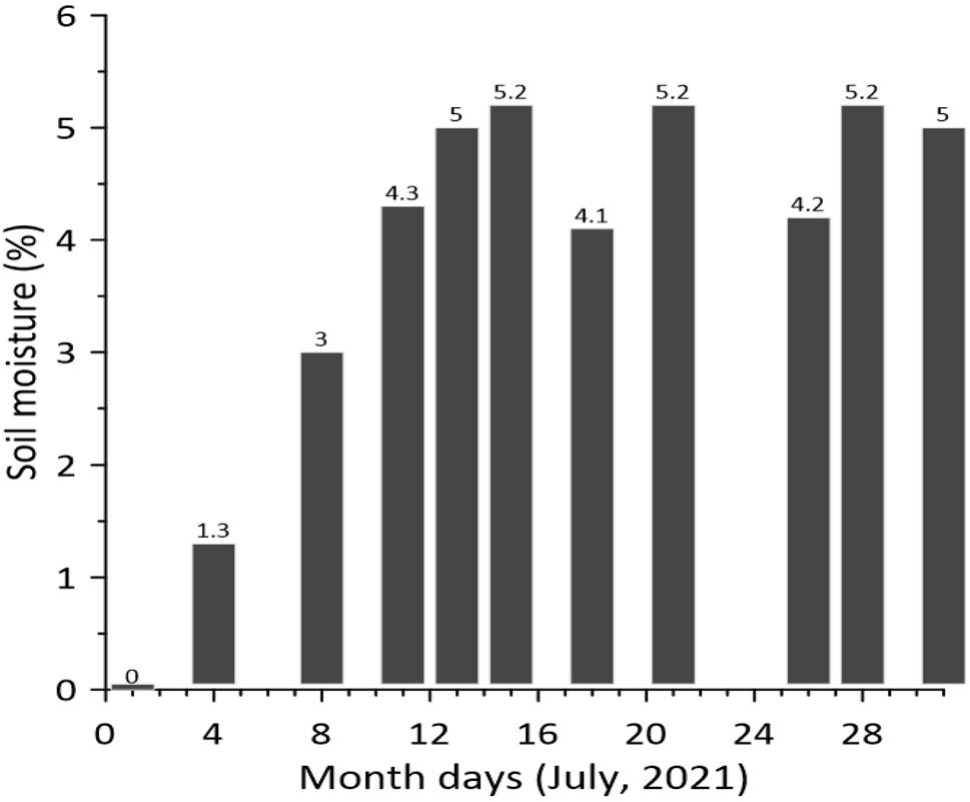
Soil moisture difference dynamics in the compared inter-windrow sites.

The difference between the soil moisture values in the compared areas appeared starting from the second measurement. The fluctuations dynamics of this difference are presented in Fig. 8.

**Figure 9:**
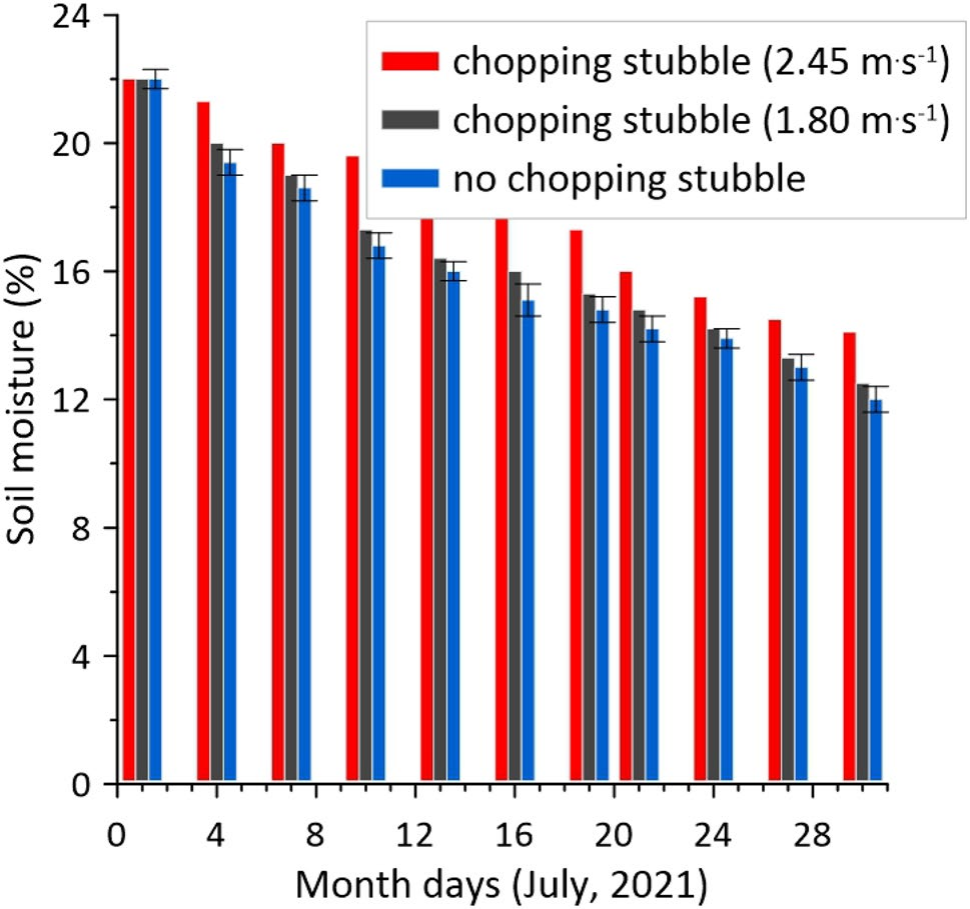
Soil moisture dynamics in the compared inter-windrow sites at different operating velocities of the harvesting unit.

Similar dynamics of soil moisture evaporation in crushed and non-crushed areas were obtained when the harvesting unit moved at different speeds (Fig. 9).

### Operational and technological estimation of machine-tractor units

Three units' operation comparative studies were carried out in the conditions of Ukraine's south: Melitopol (46° 50′ 56″ north latitude, 35° 21′ 55″ east longitude, altitude: 37 m). The harvested crop is winter wheat with a 4.5 t ha^−1^ yield. Since the unit under study is mounted, its minimum turning radius is equal to the minimum turning radius of the tractor itself. For XTZ-16131, ${R}_{\text{min}}$ value is 6.55 m. The unit worked on a field in which ${L}_{o}$ = 1470 m. The values of the other parameters included in Eq. (2) are as follows: ${B}_{p}$ = 5.90 m; $L$ = 9.80 m; $D$ = 3.80 m. With this in mind, from formula (2), we obtain that ${C}_{\text{opt}}$ = 132 m. This is equal to 23 operating passes of the harvesting unit. The operating results of the compared units are presented in Table 3.

**Table 3:** Operational and technological performance indicators of the compared units.

Index	Value for unit		
	New	Conventional	
		Harvesting	Discing
Condition and operation mode			
Operating velocity (m s^−1^)	8.3	8.4	8.0
Operating width (m)	5.91	5.92	6.8
Stubble height (cm)	15.1 $\pm$ 0.3	14.8 $\pm$ 0.5	–
Tillage depth (cm)	6.1 $\pm$ 0.4	–	7.0 $\pm$ 0.6
Performance for 1 h			
Main operating time (ha h^−1^)	4.9	5.0	5.4
Total operating time (ha h^−1^)	3.9	3.9	4.6
Specific fuel consumption (kg ha^−1^)	4.3	4.8	4.9
Operational and technological indicators			
Operating time utilization rate	0.80	0.78	0.86
Process safety factor	0.98	0.98	0.97
Stroke factor	0.84	0.83	0.86

## Discussion

### Harvesting unit's operating mode influence on the soil moisture dynamics

In the process of operating movement at an operating velocity of 2.52 m s^−1^, the harvesting unit chopped the stubble and incorporated it into the soil. The height of the cultivated stubble did not exceed 16 cm. According to research^31^, it is advisable to maintain the value of this parameter at a level of 25 cm. In this case, moisture in the soil is retained longer than with a stubble height of 8 or 75 cm.

In our case, the soil capillary structure was destroyed during unit movement, slowing soil moisture evaporation. According to research data^27^, when processing the wheat stubble background to a depth of 180–240 mm, the content of soil particles with a diameter of up to 10 mm reached 60–75%. The number of soil particles with a more than 50 mm diameter did not exceed 10%.

Generally, soil moisture value tends to decrease over time (Fig. 7). However, the intensity of this process is much lower than in the inter-windrow space without chopping stubble and soil. In the case of precipitation, soil moisture increased in both areas (zone A, Fig. 7).

As follows from the results of field studies, after 11 days, the difference in soil moisture at a depth of 7–10 cm was 4.3% in absolute terms or 15.3% in relative terms. Further, for 20 days, this difference remained almost stable, varying between 4.1 and 5.2% (Fig. 8).

As noted above, the movement velocity of the considered harvesting unit was relatively high and amounted to 2.52 m s^−1^. In several cases, its value has to be reduced due to the unevenness of the harvested field profile, crop yield, and/or the increased presence of weeds in crops. At the same time, experimental studies have established that an increase in the rate of soil disking leads to an increase in the degree of its chopping^32^. This helps to reduce the loss of productive soil moisture.

**Figure 10:**
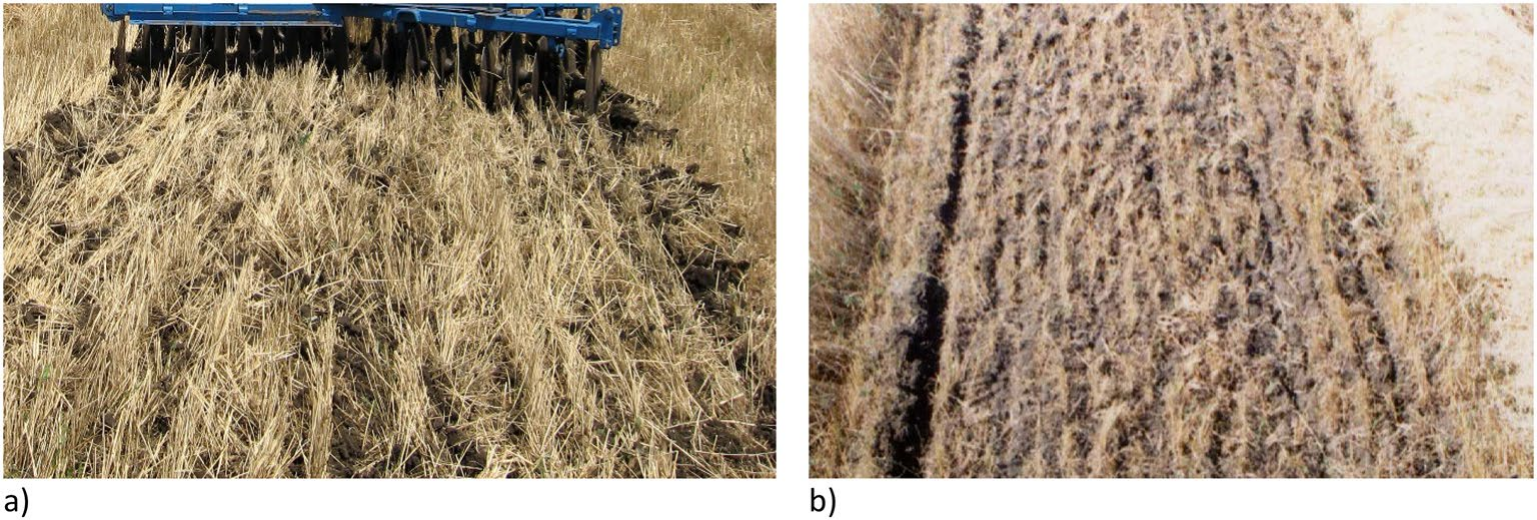
View of the disked inter-windrow sites when the harvesting unit moves at different velocities: (**a**) 1.80 m s^−1^; (**b**) 2.45 m s^−1^.

Studies of this harvesting unit operation results at two different speed modes showed the following. The degree of chopping wheat stubble and crumbling of the soil by the disc harrow increases with arising the velocity of its movement from 1.80 to 2.45 m s^−1^. The quantitative characteristics of these processes were not determined. However, their qualitative difference is easy to determine even visually (Fig. 10). As you can see, the amount of unincorporated plant residues is less in the area with a higher unit movement speed. This is consistent with the data presented in the article^33^. According to data^34,35^, a visual assessment of the field surface, in principle, has a high correlation with the yield of the crop sown on this field.

Over time, soil moisture decreased in all areas. However, starting from the 4th day of observations, the dynamics of this process was different (Fig. 9). In the area cultivated at the unit speed of 2.45 m s^−1^ (site 1), soil moisture remained higher until the end of observations. Compared to the untreated inter-windrow space (site 3), this excess was 7.5–20.5% in relative terms. When the harvesting unit moved at a velocity of 1.80 m s^−1^, the background of the field (site 2) was formed, the mean value of soil moisture which was somewhat higher than in the uncultivated area. However, statistical analysis shows that the mean soil moisture value in site 2 is almost, in most cases, within the confidence interval for the mean soil moisture value in site 3 (see Fig. 9). Hence, it follows that to reduce the loss of soil moisture during mowing of crops with simultaneous chopping of their stubble and incorporating into the soil, the harvesting unit velocity movement should be close to 2.5 m s^−1^.

This result is consistent with the data reported in the study^36^. The authors of this work have proven that the optimal quality of processing rapeseed stubble to a depth of 8 cm (in our case, 6–7 cm) occurs when the disc harrow operates at a speed of 2.5–3.0 m s^−1^.

### Operational and technological indicators of the harvesting unit

Analysis of the obtained data showed the following. The performance of all three units is approximately the same (Table 3). This means that the field of the same area when using two separate units (harvest and disc) instead of one combined (harvest-disc), will be processed approximately twice as long. Very often, this is highly undesirable from an agronomic point of view.

The second crucial comparative indicator is fuel consumption per unit of cultivated area. A combined harvesting-disc unit is equal to 4.3 kg ha^−1^. When using two units for different purposes, the total fuel consumption increases to 9.7 kg ha^−1^, i.e., it increases by 2.25 times.

The operational and technological performance of the combined harvesting-disc unit is approximately on the same level as compared. This is especially true for such an indicator as the reliability coefficient of the technological process. For the combined unit, its value is not less than that of the compared ones, and at the same time, it reaches the level of 0.98 (with its maximum value equal to 1.0).

## Conclusions

Mowing cereal crops into swathes with soil simultaneous shallow loosening in the inter-windrow space is advisable. Using a unit consisting of a reverse-conversed tractor, a front windrower, and a rear-mounted disc harrow for this purpose provides the following advantages.

1. It should be taken into account that conversion of the tractor to reverse, as well as attaching a front mower and a disc harrow to it, is carried out for a relatively long period of harvesting. The laboriousness of completing such a combined unit of 1.442 person-hours can be considered quite acceptable.
2. Mowing the crop simultaneously with chopping the stubble and incorporating it into the soil helps reduce soil moisture loss. Within a month, this decrease can reach 4.1–5.2% in absolute terms and 15–45% in relative terms. The harvesting unit velocity movement should be close to 2.5 m s^−1^ for the practical implementation of the indicated reduction in soil moisture losses.
3. Using one combined unit instead of two (harvesting and soil-cultivating) with almost the same reliability of technological operations reduces the processing time of one field by nearly two times and fuel consumption reduction per unit of the cultivated area by 2.25 times.

## Data Availability

Abstracted data is available from the corresponding author upon reasonable request.
